# Pathway Driven Target Selection in *Klebsiella pneumoniae*: Insights Into Carbapenem Exposure

**DOI:** 10.3389/fcimb.2022.773405

**Published:** 2022-01-31

**Authors:** Federico Serral, Agustin M. Pardo, Ezequiel Sosa, María Mercedes Palomino, Marisa F. Nicolás, Adrian G. Turjanski, Pablo Ivan P. Ramos, Darío Fernández Do Porto

**Affiliations:** ^1^ Instituto de Cálculo, Facultad de Ciencias Exactas y Naturales, Universidad de Buenos Aires (UBA), Buenos Aires, Argentina; ^2^ Instituto de Química Biológica de la Facultad de Ciencias Exactas y Naturales (IQUIBICEN), CONICET-Universidad de Buenos Aires, Buenos Aires, Argentina; ^3^ Facultad de Ciencias Exactas y Naturales, Departamento de Química Biológica, Universidad de Buenos Aires, Cdad. Universitaria, Buenos Aires, Argentina; ^4^ Laboratório de Bioinformática (LABINFO), Laboratório Nacional de Computação Científica (LNCC), Petrópolis, Brazil; ^5^ Centro de Integração de Dados e Conhecimentos para a Saúde (CIDACS), Instituto Gonçalo Moniz, Fundação Oswaldo Cruz (Fiocruz - Bahia), Salvador, Brazil

**Keywords:** carbapenem resistance, drug targeting, genome-scale metabolic models, *Klebsiella pneumoniae*, target selection

## Abstract

Carbapenem-resistant *Klebsiella pneumoniae* (CR-KP) represents an emerging threat to public health. CR-KP infections result in elevated morbidity and mortality. This fact, coupled with their global dissemination and increasingly limited number of therapeutic options, highlights the urgency of novel antimicrobials. Innovative strategies linking genome-wide interrogation with multi-layered metabolic data integration can accelerate the early steps of drug development, particularly target selection. Using the BioCyc ontology, we generated and manually refined a metabolic network for a CR-KP, *K. pneumoniae* Kp13. Converted into a reaction graph, we conducted topological-based analyses in this network to prioritize pathways exhibiting druggable features and fragile metabolic points likely exploitable to develop novel antimicrobials. Our results point to the aptness of previously recognized pathways, such as lipopolysaccharide and peptidoglycan synthesis, and casts light on the possibility of targeting less explored cellular functions. These functions include the production of lipoate, trehalose, glycine betaine, and flavin, as well as the salvaging of methionine. Energy metabolism pathways emerged as attractive targets in the context of carbapenem exposure, targeted either alone or in conjunction with current therapeutic options. These results prompt further experimental investigation aimed at controlling this highly relevant pathogen.

## 1 Introduction

Antibiotics are among the most valuable and successful pharmaceutical drug classes. Since the discovery of penicillin in 1928 and the subsequent widespread use of this and other compounds to treat bacterial infections, millions of deaths have been averted globally. As a result, infectious diseases are no longer the leading cause of death in the United States and other industrialized nations as they were at the dawn of the 20th century (Leading Causes of Death, 2021). Nevertheless, the phenomenon of bacterial resistance to antibiotics that, coupled to the absence of novel drug classes under development, and industry dismay in this field, has produced a scenario of limited therapeutic options to combat some pathogens. Reports in Europe alone show a substantial burden of infections with antibiotic-resistant bacteria compared to other infectious diseases, with a 2.46-fold overall increase in deaths between 2007 and 2015. (Cassini et al., 2019).

This scenario is even more challenging when infections are associated with ESKAPE bacteria (*Enterococcus faecium*, *Staphylococcus aureus*, *Klebsiella pneumoniae*, *Acinetobacter baumannii*, *Pseudomonas aeruginosa*, and *Enterobacter* spp.). These pathogens are of particular concern due to their role in infections of human organs (e.g., urinary tract and lung) and their ability to evade the action of commonly used antimicrobials (Pendleton et al., 2013). These six bacteria are in the World Health Organization’s Global Priority List of Antibiotic-Resistant Bacteria to Guide Research, Discovery, and Development of New Antibiotics (WHO, 2017). In particular, carbapenem-resistant *K. pneumoniae* (CR-KP) has attracted attention in recent years due to its rapid expansion, high mortality, and morbidity. This pathogen is already considered an epidemic in some countries (Tacconelli et al., 2018; Gonzalez-Ferrer et al., 2021). The recrudescence of the burden of bacterial infectious diseases, including CR-KP, poses a key challenge to global health. In this context, multidisciplinary efforts to disclose new bacterial targets and compounds are urgently required.

Traditional approaches to antibiotic discovery involving high-throughput screening of libraries containing thousands of compounds are now considered largely exhausted and usually lead to the repeated identification of hits. Alternative strategies include identifying microbial strains from lowly explored environments, screening new microbial taxa, mining microbial genomes, and using innovative assays, all of which have led to the identification of potential antimicrobial compounds (Donadio et al., 2010). In addition, target-based approaches have re-emerged. After an initial disappointment with genome-based studies, the availability of multiple layers of omics information for clinically relevant bacterial species have given a new impulse to this area. These approaches rely heavily on successful target selection, the pivotal challenge of early stages of drug discovery (Zuniga et al., 2015).

The sheer availability of genomic information has greatly benefited the study of microbial metabolism, allowing us to accurately depict and model the capabilities of organisms by the analysis of their genomes. Multiple efforts have been made to study the metabolism of organisms on a genomic scale, generating hundreds of reliable genome-scale metabolic models (GEMs) in the last decade (Kim et al., 2017). The number of resources and methods aimed at facilitating the draft generation, standardization, and curation of metabolic reconstructions has also bloomed (Mendoza et al., 2019). These tools can be broadly divided into two sets: those that provide curated vocabularies (or ontologies) of compounds, reactions, associated enzymes, and pathways; and software that support GEM reconstruction and modeling processes. The former group includes BiGG (King et al., 2016), KEGG (Kanehisa et al., 2014), BioCyc (Caspi et al., 2016) and Reactome (Fabregat et al., 2016). Among available tools, ModelSEED (Henry et al., 2010), Pathway Tools (Karp et al., 2011) and RAVEN (Wang et al., 2018) stand out by being widely employed in GEM reconstruction. While the generation of an initial metabolic draft for any given organism can be rapidly achieved, to produce a high-quality metabolic reconstruction, careful manual examination, and curation of the initial reconstruction must follow.

Once GEMs are produced, they can address a multitude of biological questions. By combining genomic, phylogenomic, and phenotypic analyses of a diverse set of *K. pneumoniae*, Blin et al. studied how the variability of metabolic and genomic traits impacts niche specificity and pathogenic specialization of this bacterium, effectively probing the metabolic diversity of *Klebsiella* (Blin et al., 2017). Additionally, Norsigian *et al.* constructed GEMs for 22 K*. pneumoniae* strains with varying degrees of antibiotic resistance and predicted their growth capabilities on multiple carbon, nitrogen, sulfur, and phosphorus sources, reaching a set of media components with discriminatory power to identify strains resistant to amikacin, tetracycline, and gentamicin (Norsigian et al., 2019). Our group has previously associated metabolic information during the prioritization of polymyxin-resistant *K. pneumoniae* drug targets by using an integrated, multi-omics approach that yielded 29 potential drug targets against MDR *K. pneumoniae* (Ramos et al., 2018), of which 18 were in common to those disclosed in a recent study that employed a dual gene- and network-centric strategy to identify potential targets (Cesur et al., 2020). In summary, the emerging field of systems biology provides a key framework for understanding cellular metabolism under different conditions and can be applied to facilitate the discovery of new drugs and to explore novel molecular targets. One fundamental advantage of studying the metabolic context of candidate targets is that the results are expected to allow the design of possible combined therapies by targeting more than one molecule from the same pathway (Oldfield and Feng, 2014; Sosa et al., 2018; Tyers and Wright, 2019). The synergistic effect of this type of treatments, potentially decreases the chances of resistance arising due to single-target mutations.

Here, we expand upon our previous results (Ramos et al., 2018) by directing our focus to the identification of potential targets aimed at combating *K. pneumoniae* in the context of carbapenem exposure, including CR-KP. A manually revised metabolic network for a carbapenem-resistant *K. pneumoniae* is presented. By leveraging the capabilities of this model with available omics information for *K. pneumoniae* bacteria, including expression data on carbapenem exposure and homologs shared with 39 other representatives of pathogenic *K. pneumoniae*, the pathways disclosed contain a core set of protein targets with desirable characteristics, from a drug development perspective. These targets could be used to accelerate further studies aimed at controlling this important pathogen. We envisage that our results will be particularly useful to inform initial steps of lead discovery through the identification of targets of interest.

## 2 Material and Methods

### 2.1 Bacterial Strain and Genome Annotation for the Reference Bacteria

*Klebsiella pneumoniae* Kp13, a carbapenemase (KPC-2) producer resistant to many antibiotics, was used as a reference during this work. Our group has determined its complete genome (Ramos et al., 2012; Ramos et al., 2014), which comprises one 5.3 Mbp circular chromosome and six plasmids (totaling 0.43 Mbp). This genome was previously annotated by using the SABIA pipeline (Almeida et al., 2004), predicting 5,736 coding sequences (CDSs). Original annotations and sequences for this bacterium are available at the BioProject/NCBI (https://www.ncbi.nlm.nih.gov/bioproject/) under accession no. PRJNA78291. To enrich functional annotations and increase the quality of the resulting metabolic network, an annotation pipeline based on the standard operating procedures (SOPs) of the Integrative Services for Genomic Analysis (ISGA) (Hemmerich et al., 2010) was performed as previously reported (Burguener et al., 2014; Palomino et al., 2018). The Genbank annotation file was expanded with Enzyme Commission (EC) annotations, which increased the accuracy of the gene-protein-reaction associations inferred by Pathway Tools (Karp et al., 2011).

### 2.2 Calculation of Orthologs Across Pathogenic *K. pneumoniae*

Whilst *K. pneumoniae* Kp13 was used as a reference, we also determined orthologs among 39 human pathogenic *K. pneumoniae* strains (including *K. pneumoniae* Kp13) with full-length genomes (ie. closed assemblies) available in GenBank/NCBI. This was done to focus only on broadly conserved potential targets, avoiding the ranking of proteins with highly druggable features but present only in few bacteria, such that their epidemiological relevance would need to be established. Mauve (Darling et al., 2010) was used to determine protein orthologs, and only protein pairs sharing ≥ 60% identity and ≥ 70% coverage were kept. Genomes and corresponding accession numbers used in this analysis are listed in **Supplementary Table 1**.

### 2.3 Automatic GEM Reconstruction of *K. pneumoniae* Kp13

To build the *K. pneumoniae* Kp13 metabolic network (Kp13-GEM), we used the PathoLogic module within Pathway Tools v. 18.0 environment (Karp et al., 2011). This tool uses an annotated genome in GenBank format as input to create a Pathway/Genome Database (PGDB) containing the predicted metabolic pathways of a given organism. Metabolic reconstruction included determining gene-reaction-pathway associations, which are primarily based on the corresponding EC number, as well as protein product annotations. This draft metabolic model underwent extensive manual refinement.

### 2.4 Manual Refinement of Kp13-GEM

**Figure 1:**
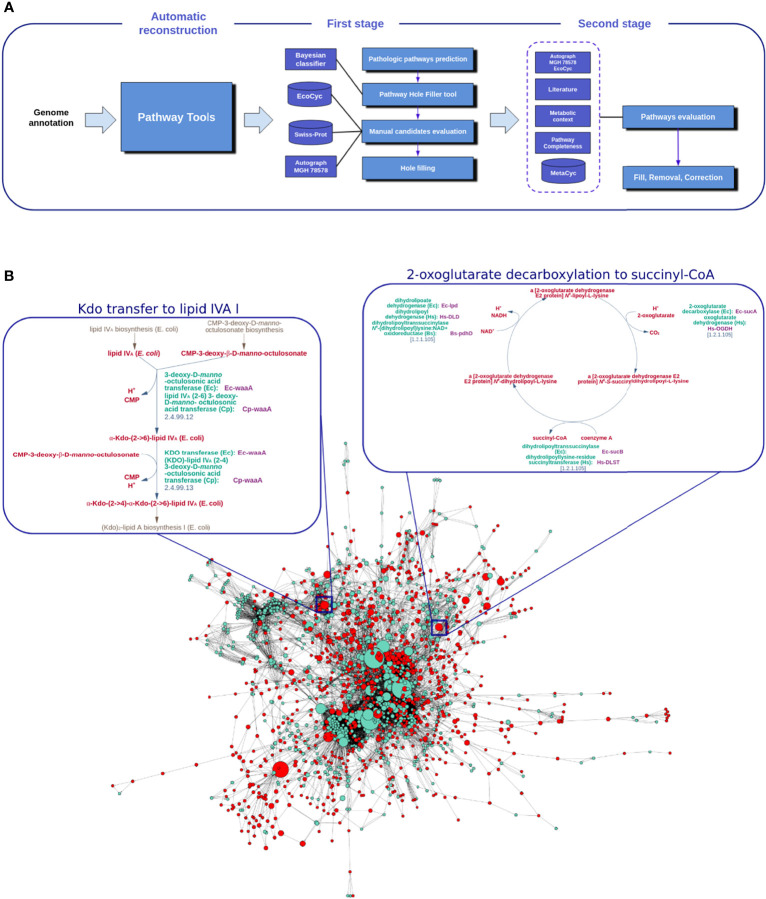
Achieving a manually refined metabolic network for *K. pneumoniae* Kp13, Kp13-GEM. In **(A)** the main steps involved in the curation process of Kp13-GEM are shown, initially by an automatic generation of a draft network model using Pathway Tools. This model is subjected to a first annotation stage, where pathway holes and candidates are manually evaluated using the Pathway Hole Filler algorithm, followed by a second stage of curation where all pathways are evaluated. Panel **(B)** shows Kp13-GEM represented as a reaction graph. Nodes represent reactions, which are linked if a product in a given reaction is used by another as a substrate. The size of the nodes is proportional to their betweenness centrality. The red nodes are choke-points. The metabolic routes for Kdo transfer to lipid IV_A_ and 2-oxoglutarate decarboxylation to succinyl-CoA, top ranked pathways in and, respectively, are detailed in the boxes next to the reaction graph.

Kp13-GEM refinement was based on two sequential steps (**Figure 1A**). First, missing reactions (holes) were determined by the Pathway Hole Filler (PHF) module within Pathway Tools. A pathway hole is a reaction for which no enzyme has been identified in the genome annotation, despite the presence of enzymes known to catalyze one or more reactions of a predicted pathway (Green and Karp, 2007). PHF not only identifies reactions of incomplete pathways but suggests gene candidates that could fulfill the corresponding enzymatic function. The candidates proposed by the PHF were evaluated using the Autograph methodology (Notebaart et al., 2006) and the extensively curated and experimentally validated metabolic network of *K. pneumoniae* MGH 78578 (Green and Karp, 2004; Liao et al., 2011) as well as the metabolic model for *Escherichia coli* MG1655, available in EcoCyc, which comprises the most complete set of reactions involved in the metabolism of this bacterium (Keseler et al., 2017). BLAST and textual searches in Swiss-Prot (http://UniProt.org) (Boutet et al., 2016) were also performed. In the second step, all pathways were analyzed to assess their degree of completeness (i.e., the ratio between the number of reactions catalyzed by a known protein-coding gene and the total number of reactions occurring in the pathway). Combined, this comprehensive strategy supported by an extensive literature search and manual annotation allowed us to complete, correct, or remove pathways as necessary.

In addition to filling pathway holes, we also evaluated and refined incomplete metabolic pathways and decided upon the removal or retention of pathway variants (alternative reaction paths leading to the same metabolic objective), allowing for their accurate representation. Biological and experimental evidence in *K. pneumoniae* or taxonomically related bacteria, as well as the previously cited pathway resources, were also used to inform these decisions, and more details of this iterative process of refining the metabolic model can be found in **Supplementary Text**. The final metabolic model was exported in System Biology Markup Language (SBML) format to facilitate further reuse.

### 2.5 Graph Representation and Identification of Choke-Point Reactions

The representation of GEMs as a graph allows the identification of nodes that may be subjected to selective pressures in the metabolic context. We used a reaction graph to represent the Kp13 metabolic network (**Figure 1B**). In this representation, nodes represent reactions, and there exists an edge between two nodes if the product of one reaction is used as a substrate on the reaction that follows (Cottret and Jourdan, 2010). Once a graph was derived, we calculated the frequency of all compounds as reaction participants using in-house Python scripts. Those that most frequently appeared as reaction participants were considered a potential currency compound (which included water, protons, ATP, and other cofactors). After manual inspection of this list, a total of 26 compounds were ruled out to avoid artificial links on the reaction graph. Cytoscape v. 3.1.0 (Shannon et al., 2003) was then used for network visualization and calculation of topological metrics, including betweenness centrality (BC). The BC of a given node *v* in Kp13-GEM is calculated as $BC(v)=\Sigma_{s\neq v\neq t\in Kp13\text{-GEM}}\frac{Q_{st}(v)}{Q_{st}}$ , where Q_st_ is the number of shortest paths between nodes *s* and *t* in the network and Q_st_(*v*) is the number of shortest paths from nodes *s* to *t* using *v* as an intermediate. High values of BC for a reaction node reflect the participation of this reaction as a mediator of many other metabolic routes, and its blockage could potentially disrupt the metabolic network by altering multiple pathways.

Choke-point analysis was conducted within Pathway Tools. Choke-point reactions are those that either uniquely produce or consume a given product or substrate, respectively (Yeh et al., 2004). In this sense, it is assumed that choke-point blockade may lead to the lack of an essential compound or the accumulation of a toxic metabolite in the cell; thus, these types of reactions have great significance in drug targeting.

### 2.6 Transcriptomic Response of *K. pneumoniae* Upon Carbapenem Exposure

To capture the transcriptomic landscape of *K. pneumoniae* bacteria exposed to carbapenems, we reanalyzed a previously published work that used RNA-seq to determine how sublethal concentrations of imipenem treatment affect *K. pneumoniae* 2 h and 24 h after carbapenem exposure compared to untreated controls (Van Laar et al., 2015). Specifically, we focused on the early time point (2 h), representing the initial response against the drug, and calculated differentially expressed genes (DEGs) compared to controls. The raw count matrix was obtained from GEO (accession number GSE62045), and after filtering out genes with low counts using the *filterByExpr* function within the edgeR package (Robinson et al., 2010), TMM-normalized expression levels were obtained. DEGs were calculated using a quasi-likelihood negative binomial model implemented in the function *glmQLFit* within edgeR, and the design matrix was constructed using biological replicates distributed over the control and 2 h exposure as experimental factors. We retained DEGs with FDR-corrected P-values less than 0.05 and log_2_-transformed fold-changes greater than 1, reasoning that genes significantly more expressed during exposure to carbapenem would be fit for drug targeting in a joint analysis with other druggability metrics (described in the following Section), thus representing potential candidates involved in the early drug response that could be synergistically targeted.

### 2.7 Data Integration and Target Prioritization

As the last step of our analysis, we classified the different metabolic pathways of Kp13-GEM using complementary scoring strategies aimed at determining which pathways are relevant to be targeted by new antimicrobial drugs capable of inhibiting broadly conserved targets across *K. pneumoniae* bacteria and synergizing with carbapenems. For this purpose, Kp13-GEM and the calculated data were integrated into Target-Pathogen, a web platform allowing multi-omics data integration aimed at prioritizing targets (available in http://target.sbg.qb.fcen.uba.ar/patho/) (Sosa et al., 2018). First, we defined a pathway as druggable if at least one of the proteins involved was predicted to be structurally druggable (Sosa et al., 2018). After ruling out non druggable pathways, we defined two scoring functions as follows to assign a score to each pathway predicted in Kp13-GEM. Equation (1) defines the importance of an entire pathway to be used as a target according to its completeness, centrality and choke-point properties of its reactions (both proxies of metabolic importance), essentiality and low identity with human proteins. Thus, for each pathway, we defined its scoring function as:


(1) $$S=\frac{C+Chk+Cy+H+Es}{5}$$

Where *C* is “completeness” (i.e., the ratio between the number of reactions of the pathway associated with a protein-coding gene and the number of reactions catalyzed by a known protein in the pathway). *Chk* is the number of choke-points in the pathway normalized by the total number of reactions present in each pathway. *Cy* is the maximum betweenness centrality among all reactions in the pathway, normalized by the reaction with maximum centrality in the entire reaction graph representation. *H*, the off-target score, reflects the results of a BLASTP search of each protein in each pathway against the human proteome database (Gencode v. 17), scaled using 1 - max(alignment identity), such that when a protein has no hit in the human proteome, the value is 1, and if it has e.g. 2 hits, with identities of 40% and 60%, the score is calculated as 0.4 (human_offtarget = 1 - 0.6, using the maximum identity). In this case, *H* is the average of the off-target score for each protein in the pathway. Finally, *Es* is the proportion of essential genes in the pathway.

We also sought to incorporate in our prioritization scheme a set of criteria aimed at identifying candidate targets that could be used in a synergic manner for the control of carbapenem-resistant *K. pneumoniae*. Equation (2) expresses this strategy:


(2) $$S=\frac{(C+Chk+Cy+H+Es)/5+Cp}{2}$$

where, in addition to the parameters described for Eq. 1, a term denoted by *Cp* represents the proportion of proteins overexpressed during carbapenem exposure (detailed in the previous Section).

### 2.8 Availability of Materials and Data

All the data generated and integrated in this study, including metabolic annotations and related meta-data, are openly available at the Target-Pathogen web server interface, and can be reached at <http://target.sbg.qb.fcen.uba.ar/patho/genome/Kp13>. The metabolic model for *K. pneumoniae* Kp13 (Kp13-GEM) in SBML format is available in **Supplementary Data**.

## 3 Results and Discussion

### 3.1 *K. pneumoniae* Kp13 Metabolic Network Refinement

We performed a whole-genome-based reconstruction of the Kp13 metabolic network (Kp13-GEM) using Pathway Tools followed by manual curation. The automatic reconstruction of Kp13-GEM with Pathway Tools resulted in a draft metabolic model composed of 386 metabolic pathways, 2,223 reactions (1,984 with enzymatic activities and 55 with transport function), and 1,714 compounds. A total of 1,554 genes/proteins were assigned to the 1,984 enzymatic reactions that compose the Kp13-GEM. We identified 141 pathway holes (reactions without associated genes) in this draft model, which were distributed across 98 metabolic pathways with different degrees of completeness. Refinement of Kp13-GEM began by manually inspecting the draft GEM using the Pathway Hole Filler (PHF) algorithm. Based on this tool, 41 proteins could be assigned to their respective reaction by filling the holes. During this process, reactions corresponding to 57 metabolic pathways were evaluated, and 26 could be completed (i.e., all their reactions had a gene assigned). In the last refinement step, using the Autograph methodology and bibliographic evidence, 6 pathways were added, 2 were corrected (changed to alternative pathways) and 20 were removed after being identified as false positives during manual curation. The summary of each step of the procedure is found in **Supplementary Table 2**, while in **Supplementary Table 3** we present details of the metabolic pathways that were modified and the corresponding evidence that substantiated these actions.

Next, we compared Kp13-GEM with other microbial genome-scale reconstructions available in BioCyc. GEMs in the database present variable annotation quality, divided into Tier 1 (extensive manual refinement), Tier 2 (limited manual refinement), and Tier 3 (fully automatic reconstructions). **Table 1** shows key characteristics of Kp13-GEM compared to eight other models, including that of three *Klebsiella* species. Kp13-GEM presents 386 pathways, 2,175 reactions, 1,997 assigned enzymes, and 1,701 compounds. Compared to *E. coli* (EcoCyc), the richest manually curated model available to date for any single microbe (Keseler et al., 2021), Kp13-GEM has an increased number of pathways and enzymatic reactions, which could reflect not only the larger genome size of *K. pneumoniae* Kp13 (5.74 Mb) compared to *E. coli* MG1655 (4.64 Mb), leading to the encoding of more biological functions in the former but also the possibility that false-positive pathways remained in the model even after manual revision. The *E. coli* model, however, presents a much larger number of transport reactions, reflecting the profound annotation effort put in this model, particularly considering that, in general, automatic genome annotations fail to identify the substrates of most proteins with transport activity (Krummenacker et al., 2019). The number of compounds in the *E. coli* model was also higher (2,616 versus 1,701 in Kp13-GEM), probably as a result of accounting for many specialized metabolic functions displayed by this versatile bacterium, again in line with the high quality of this model’s annotation, which accumulates the equivalent of decades of human effort and literature reconciliation. When compared to models with lower annotation quality in BioCyc, such as Tier 2 and Tier 3, Kp13-GEM presents a higher number of enzymes assigned to reactions, totaling 1,997, while the range for Tier 2 databases was 929-1,111 and of 1,343-1,537 for those in Tier 3 (**Table 1**). This result indicates that a high fraction of metabolic reactions have an associated gene instead of presenting as orphan reactions. Correct and complete associations between reactions and genes are paramount to downstream uses of any metabolic model. Such is the case of target prioritization, our focus in the following sections.

**Table 1:** Key characteristics of the metabolic reconstruction of *K. pneumoniae* Kp13 compared to other models produced using the BioCyc ontology.

Organism and BioCyc annotation level, when applicable	Genome size (Mb)	No. of pathways	Enzymatic reactions	Transport reactions	No. of enzymes	No. of compounds
**This work**						
*K. pneumoniae* Kp13	5.74	386	2,175	55	1,997	1,701
**BioCyc Tier 1**						
*E. coli* MG1655	4.64	338	1,799	480	1,555	2,616
**BioCyc Tier 2**						
*Acinetobacter baumannii* ATCC 17978	4.02	255	1,354	76	929	1,123
*Bacillus subtilis* 168	4.21	273	1,505	92	1,067	986
*E. coli* EDL933	5.53	284	2,290	511	1,670	1,472
*Mycobacterium tuberculosis* H37Rv	4.41	243	1,728	74	1,163	1,930
*Shigella flexneri* 2457T	4.6	267	1,467	141	1,111	1,086
**BioCyc Tier 3**						
*K. oxytoca* 10-5245	6.18	385	1,802	220	1,537	1,334
*K. pneumoniae* HS11286	5.68	404	2,020	75	1,356	1,507
*K. pneumoniae* MGH 78578	5.69	403	1,971	76	1,343	1,502

Mb, megabases.

### 3.2 Leveraging Kp13-GEM to Score *K. pneumoniae* Pathways and Find Novel Molecular Targets for Combined Drug Therapies

Once the refinement of Kp13-GEM was finished, we sought to score entire pathways (rather than single proteins) to explore novel molecular targets. We reasoned that when combined therapies are used, targeting more than one protein of the same pathway or yet multiple pathways would lower the chances of resistance development, and once it does, the impact on therapeutic outcome may be decreased (León-Buitimea et al., 2020). We applied an *a priori* filter to discard proteins for which we could not obtain a representative structural model that harbored at least one druggable pocket. A total of 347 candidate druggable pathways were disclosed using this strategy, representing 89.9% of identified pathways in Kp13-GEM. This massive target space still requires further narrowing, and for this, we took into account essentiality, off-target and metabolic criteria. **Table 2** shows the top 15 pathways that were kept after this procedure. Next, we specifically focused on some of these pathways.

**Table 2:** Top 15 best ranked metabolic pathways according to completeness, number of choke-points, essentiality, centrality and human off-targets, in which the last column represents the composite scoring scheme detailed in Eq. 1.

Rank	Metabolic pathway	NRx	C	Chk	Cy	E	H	Score
1	Kdo transfer to lipid IVA I	2	1	1	0.42	1	1	0.884
2	phosphatidylethanolamine biosynthesis I	2	1	1	0.35	1	0.73	0.816
3	lipid IVA biosynthesis	6	1	1	0.04	1	0.83	0.774
4	preQ0 biosynthesis	4	1	1	0.06	1	0.76	0.764
5	lipoate biosynthesis and incorporation III	3	1	1	0.42	0.67	0.72	0.762
6	pyrimidine deoxyribonucleotide phosphorylation	4	1	1	0.04	1	0.75	0.758
7	S-methyl-5’-thioadenosine degradation I	2	1	1	0.15	0.5	1	0.73
8	peptidoglycan biosynthesis I (meso-diaminopimelate containing)	10	1	0.7	0.17	0.75	1	0.724
9	flavin biosynthesis I (bacteria and plants)	9	1	0.56	0.21	0.89	0.93	0.718
10	glycine betaine biosynthesis I (Gram-negative bacteria)	2	1	1	0.05	1	0.52	0.714
11	choline degradation I	2	1	1	0.05	1	0.52	0.714
12	tetrapyrrole biosynthesis I (from glutamate)	6	1	0.83	0.13	0.86	0.74	0.712
13	glutathione biosynthesis	2	1	1	0.02	0.5	1	0.704
14	acyl carrier protein metabolism	2	1	1	0.01	0.5	1	0.702
15	lipoate biosynthesis and incorporation I	2	1	0.5	0.42	1	0.48	0.7

NRx, number of reactions; C, pathway completeness; Chk, fraction of choke-points; Cy, maximum reaction betweenness centrality in Kp13-GEM; E, fraction of essential reactions; H, fraction of off-target orthologs in human.

#### 3.2.1 LPS Biosynthesis

**Figure 2:**
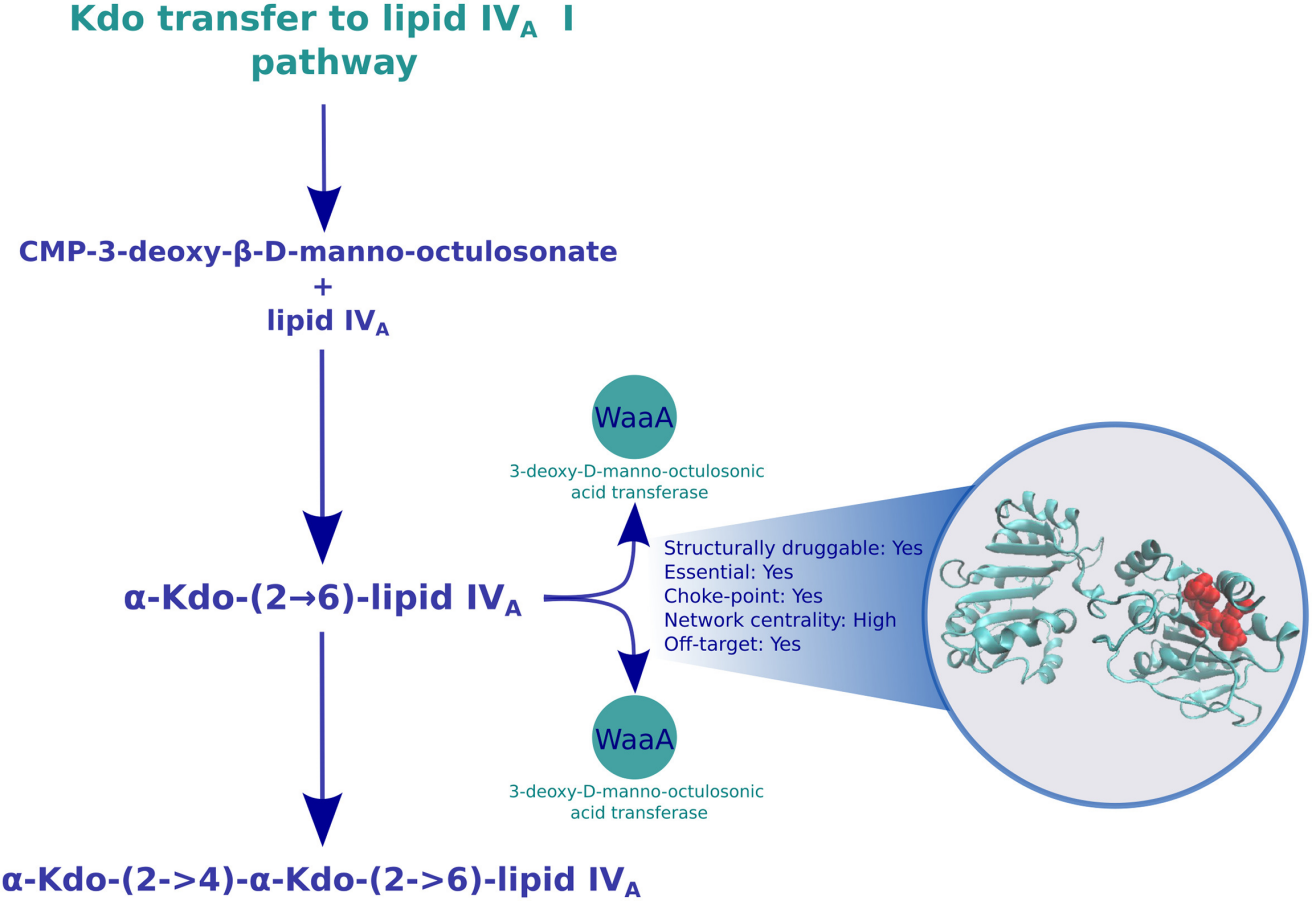
Druggable features of the Kdo transfer to lipid IV_A_ pathway. The structure of the 3-deoxy-D-manno-octulosonic acid transferase in the pathway is shown in cyan, with the predicted binding sites in red. The box next to the structure shows characteristics of this enzymes, such as druggability, essentiality, centrality in the reaction graph, and whether it is found overexpressed during carbapenem exposure.

Among the best ranking pathways, we identified LPS biosynthesis, with LPS being a major component of the Gram-negative outer membrane. LPS can be conceptually divided into three structural moieties: Lipid A, core oligosaccharides, and the O antigenic polysaccharide. The biosynthesis of lipid A begins in the cytosol with the involvement of the enzymes LpxA, LpxB, LpxC, and LpxD through the Raetz pathway (Raetz et al., 2009). Despite their potential, the technical challenges of working with enzymes that form the Raetz pathway must also be overcome (Joo, 2015). Subsequently, lipid IV_A_ is glycosylated with two 3-deoxy-D-manno-oct-2-ulosonic acid (Kdo) residues, which are added by the WaaA enzyme to produce Kdo2-lipid IV_A_. We and others have previously identified the potential of lipid A-producing enzymes (expressed from the *lpx* locus) as attractive molecular targets (Clements et al., 2002; Williams et al., 2006; Buetow et al., 2007; Erwin, 2016; Ramos et al., 2018; Ahmad et al., 2019), and multiple patents have been filed to protect intellectual properties on molecules with LpxC inhibitory and antibacterial activities (Kalinin and Holl, 2017). While we previously showed that LpxC and LpxD were high-value targets due to their broad conservation and overexpression during exposure to polymyxin B, a ‘last-resort’ antibiotic (Ramos et al., 2018), here, we put forward the potential for novel drug development involving the glycosylation function to lipid IV_A_ by targeting the *waa* operon (part of the Kdo transfer to lipid IV_A_ pathway; **Figure 2**), an idea only peripherally explored for *K. pneumoniae* (Patro et al., 2019). Since LPS biosynthesis was identified as a broadly conserved pathway target, we inferred that it would also be appropriate to be prioritized against colistin-resistant species. Although we have not found any transcriptomic experiment of *K pneumoniae* exposed to colistin, obtaining these data could be also important in order to propose specific colistin-resistant *K. pneumoniae* drug targets, since resistance against this antibiotic also poses an emerging threat to antibiotic security worldwide (Petrosillo et al., 2019).

#### 3.2.2 PreQ_0_ Biosynthesis

Another interesting, prioritized pathway is preQ_0_ biosynthesis. PreQ_0_ serves as a precursor of the most important 7-deazaguanine, queuosine (Q). Q is a modified nucleoside that is present in certain tRNAs at the wobble position of anticodons in tRNAs for Asn, Asp, Tyr, and His. The *de novo* biosynthesis of Q is exclusive to eubacteria, and humans have to obtain it from the diet or commensal bacteria (Yuan et al., 2019). PreQ_0_ is produced through four reactions from the nucleotide GTP. The first step of the pathway starts with the transformation of GTP to 7,8-dihydroneopterin 3’-triphosphate by FolE (druggability score [DS] = 0.51), which we also previously identified as an attractive target (Ramos et al., 2018), an enzyme that is also involved in the biosynthesis of 5,6,7,8-tetrahydrofolate, a crucial intermediate in the biosynthesis of nucleic acids and proteins. Subsequently, 7,8-dihydroneopterin 3’-triphosphate is converted to 6-carboxy-5,6,7,8-tetrahydropterin, and this reaction is catalyzed by QueD, a highly druggable enzyme (DS = 0.78). The latter intermediate is converted to 7-carboxy-7-deazaguanine by QueE (DS = 0.54). Finally, QueC catalyzes the conversion of the final product, preQ_0_ in an ATP-dependent manner (McCarty et al., 2009).

#### 3.2.3 Lipoylation

Two pathways associated with lipoate biosynthesis have been proposed as candidate druggable routes. Lipoate is required as a crucial cofactor by several enzyme complexes in oxidative metabolism. In bacteria, five lipoate-dependent multienzyme systems have been described: a glycine cleavage system, an acetoin dehydrogenase system and three α-ketoacid dehydrogenase complexes (pyruvate dehydrogenase, α-ketoglutarate dehydrogenase and branched-chain α-ketoacid dehydrogenase) (Spalding and Prigge, 2010). Lipoate synthase (LipA) is involved in the final step of *de novo* lipoate biosynthesis. LipA, a highly druggable enzyme (DS = 0.78), is present in both pathways and catalyzes the conversion of octanoate to lipoate. The finding that disruption of lipoylation pathways in *Mycoplasma hyopneumoniae* leads to bacterial growth inhibition (Jin et al., 2021), coupled with the particularities displayed by the prokaryotic pathway (Cao et al., 2018), strengthens the evidence that proteins involved in this process can be further prospected as novel targets against *K. pneumoniae*.

#### 3.2.4 Methionine Salvaging

The S-methyl-5’-thioadenosine (MTA) degradation pathway also appeared top-ranked (**Table 2**). MTA is generated as a side compound during polyamine biosynthesis and other important compounds. Microorganisms need to regulate MTA levels to avoid the inhibition of polyamine biosynthesis and transmethylation reactions by this byproduct. The most common way of scavenging MTA is through the widely conserved methionine salvage pathway. However, the initial processes to convert MTA to S-methyl-5-thio-α-D-ribose 1-phosphate differ between microorganisms and eukaryotic cells (Cornell et al., 1996; Sekowska et al., 2004). MTA/SAH nucleosidase, MtnK (DS = 0.44), and MTR kinase, MtnN (DS = 0.69), two druggable enzymes involved in the early steps of the pathway, emerge as attractive targets for the development of drugs aimed at selectively inhibiting microbial methionine salvage metabolism. Their disruption would lead to the buildup of MTA and S-adenosylhomocysteine, affecting the major microbial cellular functions. The antimicrobial properties of nucleosidase inhibitors, both natural and synthetic, have been studied, and their potential as broad-spectrum antibiotics against Gram-positive and Gram-negative bacteria has been underlined (Lee et al., 2004; Serpi et al., 2016; Chakraborti et al., 2020; Cornell et al., 2020). Additionally, the structural diversity in the active site of prokaryotic MtnK and the mammalian MTA phosphorylase, responsible for MTA breakdown in humans, offers an opportunity to achieve substrate specificity, potentially reducing off-targets (Serpi et al., 2016).

#### 3.2.5 Flavin Biosynthesis

Flavin biosynthesis resulted in another well-ranked pathway (**Table 2**). Riboflavin (vitamin B_2_), the direct precursor of essential cofactors FAD (flavin adenine dinucleotide), and FMN (flavin mononucleotide), plays an important role in reactions involving redox centers, including redox homeostasis, energetic metabolism and protein folding. Six (RibA [DS = 0.74]; RibD [DS = 0.94]; YigB [DS = 0.86]; Yigl [DS = 0.78]; RibE [DS = 0.81]; RibF [DS = 0.85]) of the nine enzymes that participate in this metabolic pathway presented a highly druggable score. Of note, this pathway is lacking in mammals, which must obtain riboflavin exogenously. Disruption of the microbial riboflavin biosynthetic pathway, particularly by targeting riboflavin synthase (RibE) and lumazine synthase (RibH; for which we could not infer DS due to lack of structural model), has been achieved using chemically produced inhibitors of these enzymes, which were also developed as prospective anti-tuberculosis agents (Mack and Grill, 2006; Zhao et al., 2009; Long et al., 2010). More recently, a high-throughput screening assay to disclose leads against *Brucella* spp. riboflavin synthase identified, out of 44,000 highly diverse compounds presenting drug-like properties, ten low molecular weight molecules with 50% inhibitory concentrations in the low micromolar range, of which two proved to be highly efficient in reducing viable intracellular bacteria during experimental infection assays (Serer et al., 2019).

#### 3.2.6 Glycine Betaine Biosynthesis

Glycine betaine, a well-known osmolyte that acts as an osmoprotectant, can either be obtained from medium or by *de novo* synthesis. In *K. pneumoniae*, the pathway leading to glycine betaine starts from a choline precursor and is then converted to the toxic intermediate betaine aldehyde through the druggable enzyme choline dehydrogenase (BetA; DS = 0.55). The reaction that follows, which we identified as a choke-point, is catalyzed by betaine aldehyde dehydrogenase (BetB; DS = 0.53), converting betaine aldehyde into glycine betaine. In *Pseudomonas aeruginosa*, deletion of *betBA* resulted in decreased bacterial survival in a mouse lung model, although the mechanism generating the mutant survival defect was not explored (Wargo, 2013). Since disruption of BetB would lead to the buildup of toxic betaine aldehyde, this could be a possible mechanism explaining this observation and points to the prioritization of inhibitors targeting BetB over BetA. Indeed, disulfiram metabolites (used to treat alcohol dependence in humans) have been shown to inhibit *P. aeruginosa* BetB (Velasco-García et al., 2006; Zaldívar-Machorro et al., 2011) while also displaying potent bactericidal activities against *M. tuberculosis* (Horita et al., 2012; Dalecki et al., 2015). Taken together, these results cast light on the feasibility of targeting this biosynthesis pathway, either by novel lead discovery or through the repurposing of existing drugs, such as disulfiram derivatives. Further experimental efforts are required to determine the suitability of both strategies against *K. pneumoniae*.

#### 3.2.7 Rediscovered Pathways: Production of Phosphatidylethanolamine, Peptidoglycan and Ribonucleotides

As expected, our current prioritization strategy also reidentified several pathways that we previously associated with harboring promising targets (Ramos et al., 2018), suggesting that genome-based approaches also suffer from the rediscovery problem well known for large-scale screening approaches (Genilloud et al., 2011). For instance, *pssA* (DS = 0.86), which is involved in the biosynthesis of phosphatidylethanolamine, the major phospholipid of membranes; thymidylate kinase (*tmk* gene product; DS = 0.58), an enzyme that participates in pyrimidine deoxyribonucleotide phosphorylation; and the gene products of *murF* (DS = 0.76), *murG* (DS = 0.8), *murD* (DS = 0.71) and *murE* (DS = 0.85), which participate in peptidoglycan biosynthesis, were rediscovered in the current work, and their potential is discussed elsewhere (Ramos et al., 2018). Nonetheless, this agreement reinforces the plausibility that these molecules can be developed as novel *K. pneumoniae* targets and, in addition to the druggable pathways identified here, provides an expanded molecular target space that can be further explored.

### 3.3 Incorporating Carbapenem Expression Data to Detect Druggable Pathways During Carbapenem Exposure

Next, we sought to incorporate in our scoring function a term related to expression upregulation when *K. pneumoniae* is exposed to carbapenems. For this, we reanalyzed previously generated data (Van Laar et al., 2015). The rationale behind this strategy involves appreciating that multiple bacterial cellular processes appear upregulated upon antibiotic exposure, both to counter and repair the direct damage of these drugs (such as membrane degradation) but also as a result of compensatory protein expression (Henry et al., 2015; Van Laar et al., 2015; Ramos et al., 2016; Mmatli et al., 2020; Rahim et al., 2020). In this sense, if we know which pathways are overexpressed after carbapenem exposure (probably related to the maintenance of homeostasis), we might be able to inhibit them with novel drugs resulting in a synergistic bactericidal effect. Hence, some of these upregulated proteins could be exploited using combination therapies alongside carbapenems. The usage of antibiotics with nonantibiotic activity-enhancing compounds has been proposed as an innovative strategy to address the emergence of resistant pathogens (Tyers and Wright, 2019).

#### 3.3.1 Energy Metabolism

Our results show that molecules involved in energetic metabolism emerge as promising targets in the context of carbapenem exposure (**Table 3**), while targets previously proposed to combat polymyxin resistant strains were mainly related to the synthesis of membrane components (Ramos et al., 2018) Out of the top 15 prioritized pathways, 13 were related to energy metabolism (**Table 3**). *K. pneumoniae* exposure to carbapenems induces a complex transcriptional response that involves energy metabolism (Van Laar et al., 2015; Khan et al., 2017; Sharma et al., 2019). Multiple metabolites enter the TCA pathway, such as byproducts of putrescine, arginine, proline, and phenylethylamine degradation processes (Adams-Sapper et al., 2018), and pathways that catabolize these compounds were highly-ranked in our results (**Table 3**). In the phenylethylamine degradation pathway, two highly druggable proteins are present, phenylethylamine oxidase (TynA; DS = 0.73) and phenylacetaldehyde dehydrogenase (FeaB; DS = 0.94). Both participate in choke-point reactions and present low identity with human proteins, despite being unorthodox candidates from a drug development perspective. This is because these reactions allow microbial growth using 2-phenylethylamine as a carbon source and under poor nutritional conditions, yielding H_2_O_2_ as a byproduct that, in principle, could favor intracellular stress and damage (Ravindra Kumar and Imlay, 2013), such that the value of inhibiting this function is unclear. However, the periplasmic location of hydrogen peroxide-producing TynA leads to leakage of this metabolite to the external milieu and has been proposed as a mechanism to compete with other bacteria (Elovaara et al., 2015). Additionally, in *E. coli* this protein is able to use human granulocytes as a substrate, but it is unknown whether this confers any advantages during human infection (Elovaara et al., 2015).

**Table 3:** Top 15 best ranked metabolic pathways according to completeness, number of choke-points, essentiality, centrality, human off-targets and carbapenem over-expression, in which the last column represents the composite scoring scheme detailed in Eq. 2.

Rank	Metabolic pathway	NRx	C	Chk	Cy	E	H	CP	Score
1	2-oxoglutarate decarboxylation to succinyl-CoA	3	1	1	0.03	1	0.56	1	0.859
2	phenylethylamine degradation I	2	1	1	0.03	0	0.67	1	0.770
3	trehalose biosynthesis I	3	1	0	0.19	0	1	1	0.719
4	putrescine degradation I	2	1	0.5	0.01	0	0.65	1	0.716
5	proline degradation	3	0.67	0.33	0	0	0.69	1	0.669
6	D-arginine degradation	4	0.25	0	0.03	0.5	0.89	1	0.666
7	citrulline degradation	2	0.5	0	0.15	0	0.63	1	0.628
8	S-methyl-5’-thioadenosine degradation I	2	1	1	0.15	0.5	1	0.5	0.615
9	phenylacetate degradation I (aerobic)	9	1	0.67	0.19	0	0.71	0.6	0.556
10	TCA cycle I (prokaryotic)	15	1	0.07	0.32	0.37	0.72	0.59	0.544
11	L-threonine degradation III (to methylglyoxal)	3	0.67	0.33	0.03	0.33	0.72	0.67	0.542
12	glyoxylate cycle	6	1	0	0.38	0.29	0.77	0.57	0.53
13	autoinducer AI-2 biosynthesis I	5	0.6	0.6	0.07	0.5	1	0.5	0.527
14	purine deoxyribonucleosides degradation I	4	1	0.75	0.03	0	0.83	0.5	0.511
15	catechol degradation to β-ketoadipate	4	1	0.5	0.1	0	1	0.5	0.510

NRx, number of reactions; C, pathway completeness; Chk, fraction of choke-points; Cy, maximum reaction betweenness centrality in Kp13-GEM; E, fraction of essential reactions; H, fraction of off-target orthologs in human; CP, fraction of genes in each pathway over-expressed during carbapenem exposure.

**Figure 3:**
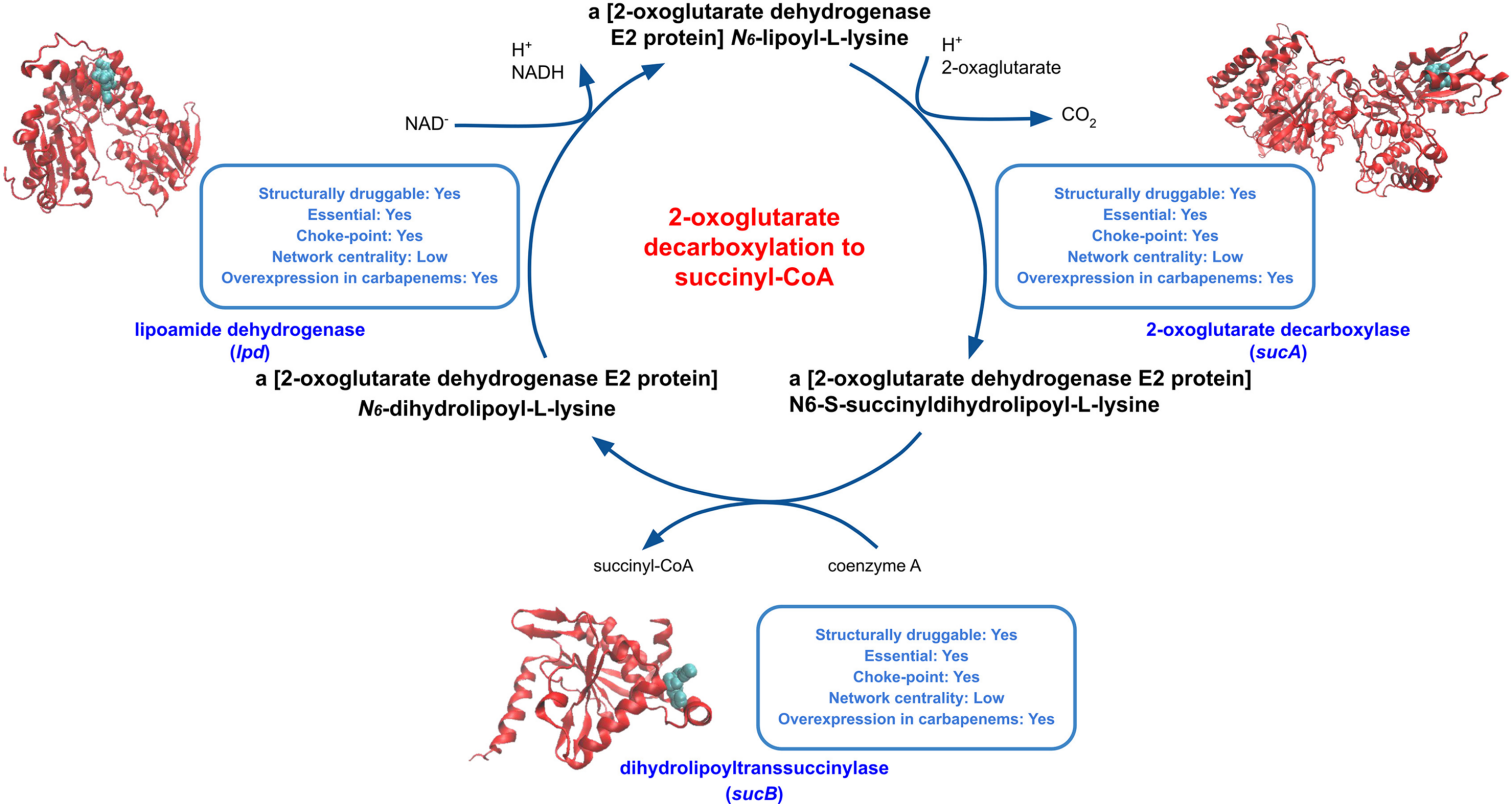
Druggable features of the 2-oxoglutarate decarboxylation to succinyl-CoA pathway. The modeled structures of enzymes in the pathway are shown in red, with the predicted binding sites in cyan. The boxes next to each structure shows characteristics of these enzymes, such as druggability, essentiality, centrality in the reaction graph, and whether it is found overexpressed during carbapenem exposure.

The 2-oxoglutarate decarboxylation to succinyl-CoA pathway harbors reactions catalyzed by the 2-oxoglutarate dehydrogenase complex (SucA [DS = 0.87], SucB [DS = 0.55] and LpdA [DS = 0.53]), a key enzyme of the prokaryotic TCA cycle (**Figure 3**). These proteins were found to be druggable and essential and were all overexpressed when *K. pneumoniae* was exposed to carbapenem antimicrobials. Moreover, 2-oxoglutarate dehydrogenase subunits play a role in the cellular response to acute stress exposure (Graf et al., 2013), making these enzymes attractive, novel and poorly explored targets. In particular, LpdA was proposed as a potential target for drug development (Deb et al., 2002). Interestingly, proline and arginine degradation pathways, which also ranked well in our prioritization (**Table 3**), produce L-glutamate as an end product, which in turn is converted into 2-oxoglutarate.

TCA and glyoxylate cycles were also highly ranked (**Table 3**), confirming the importance of energetic metabolism during *K. pneumoniae* exposure to carbapenems. Both pathways harbor druggable and essential proteins that are also associated with reactions with elevated betweenness centrality in Kp13-GEM. For example, isocitrate lyase (AceA), a highly druggable (DS = 0.92) and essential protein, which was validated as an antituberculosis target (Sharma et al., 2013; Nandakumar et al., 2014; Shukla et al., 2018). Moreover, this protein is broadly conserved in *K. pneumoniae* but does not have close homologs in the human genome, making it attractive to exploration in further experimental studies.

#### 3.3.2 Putrescine and D-Arginine Degradation

Putrescine and arginine degradation pathways also appeared in our prioritization (**Table 3**). The first pathway is catalyzed by two druggable enzymes, putrescine aminotransferase (DS = 0.6) and γ-aminobutyraldehyde dehydrogenase (DS = 0.74), which convert putrescine into γ-aminobutyrate (GABA), then metabolized *via* the GABA shunt bypassing two steps of the TCA cycle. Because they can induce apoptosis and inhibit cell growth, the levels of polyamines such as putrescines must be tightly regulated (Wallace et al., 2003). It was reported that putrescine catabolism constitutes a metabolic response to several stresses in *E. coli* (Schneider et al., 2013). The structures of some of these enzymes from *E. coli* and *Pseudomonas* have been solved (Cha et al., 2014; Wilding et al., 2017).

D-arginine catabolism was also highly ranked due to the presence of predicted essential proteins, low fraction of off-targets, and overexpression of all genes in the pathway during exposure of *K. pneumoniae* to carbapenems (**Table 3**). Despite the importance of arginine utilization in bacteria, adding to their metabolic versatility, the relevance of members of this pathway as targets is still unclear, especially given the redundancy of this process mediated by multiple pathways (Yang and Lu, 2007).

#### 3.3.3 Trehalose Synthesis

Trehalose biosynthesis is among the few highly ranked pathways unrelated to energy metabolism (**Table 3**). Bacteria can synthesize large amounts of this disaccharide to protect the integrity of the cell against a variety of environmental injuries (Argüelles, 2000), and targeting enzymes involved in this process has been extensively studied for *M. tuberculosis* (Thanna and Sucheck, 2016), particularly informed by the absence of these proteins in vertebrates. Trehalose-6-phosphate synthase (OtsA; DS = 0.71) and trehalose-6-phosphate phosphatase (OtsB; DS = 0.86) participate in this process and are conserved across a range of clinically relevant species (Cross et al., 2017), fueling recent high-throughput screening efforts that led to the discovery of specific inhibitors of these proteins (Cross et al., 2019).

## 4 Conclusions

By generating and making extensive use of a refined genome-scale metabolic network for carbapenem-resistant *K. pneumoniae* (Kp13-GEM), we prioritized pathways harboring attractive properties from a drug development perspective. Members of these pathways could be developed as candidates in further experimental studies aimed at identifying novel compounds to control *K. pneumoniae*, in particular during exposure to carbapenems. Other uses of Kp13-GEM include topology-based analyses on the network by inducing a reaction graph from the SBML-formatted model, as we have performed. Then, network theory methods can be applied, and topological metrics can be calculated to study relevant aspects of the *K. pneumoniae* metabolism, compared to those of other bacteria. Our integrative approach, where multiple layers of *omics* information were overlaid, disclosed a series of candidate pathways to inform target selection in antibiotic drug discovery, with energy metabolism emerging as a dominant biological theme warranting further consideration in the molecular target space of *K. pneumoniae.*

## Data Availability Statement

The datasets presented in this study can be found in online repositories. The names of the repository/repositories and accession number(s) can be found in the article/**Supplementary Material**.

## Author Contributions

DF, MN, and PR conceived the study design. AP, AT, DF, ES, FS, MN, MP, and PR contributed tools and performed data analysis. AP, DF, MN, MP, and PR drafted the manuscript with input from the other authors. All authors contributed to the article and approved the submitted version.

## Funding

MN was supported by a fellowship from CNPq (process no. 306894/2019-0) and grant by CAPES (process no. 88887.368759/2019-00). CONICET supported fellowships to AP and FS. ES, AT, MP, and DF are members of CONICET. This work was supported by Agencia Nacional de Promoción Científica y Tecnológica (ANPCyT, PICT-2019-01359 to DF) and Universidad de Buenos Aires (20020190200275BA to DF).

## Conflict of Interest

The authors declare that the research was conducted in the absence of any commercial or financial relationships that could be construed as a potential conflict of interest.

## Publisher’s Note

All claims expressed in this article are solely those of the authors and do not necessarily represent those of their affiliated organizations, or those of the publisher, the editors and the reviewers. Any product that may be evaluated in this article, or claim that may be made by its manufacturer, is not guaranteed or endorsed by the publisher.
