# Supplementary material for: Pathway Driven Target Selection in Klebsiella pneumoniae: Insights Into Carbapenem Exposure
Source: Front Cell Infect Microbiol. 2022 Jan 31;12:773405. doi: 10.3389/fcimb.2022.773405 (PMC8841789; doi:10.3389/fcimb.2022.773405)
Supplement: Supplementary file 1 [file DataSheet_1.pdf]

Supplementary Text

Pathway-driven target selection in carbapenem-resistant *Klebsiella pneumoniae*

Federico Serral[1#], Agustin M Pardo[1#], Ezequiel Sosa[3], María Mercedes Palomino[2,3], Marisa F Nicolás[4], Adrian G Turjanski[2,3], Pablo Ivan P Ramos[*5†], Darío Fernández Do Porto[*1,2†]

[1]Instituto de Cálculo, Facultad de Ciencias Exactas y Naturales, Universidad de Buenos Aires (UBA), Buenos Aires, Argentina

[2]Facultad de Ciencias Exactas y Naturales, Departamento de Química Biológica, Universidad de Buenos Aires, Cdad. Universitaria, Pabellón II, 4 piso, Lab QB40, C1428EGA, CABA, Buenos Aires, Argentina.

[3]Instituto de Química Biológica de la Facultad de Ciencias Exactas y Naturales (IQUIBICEN), CONICET-Universidad de Buenos Aires, Buenos Aires, Argentina.

[4]Laboratório de Bioinformática (LABINFO), Laboratório Nacional de Computação Científica (LNCC), Petrópolis, Brazil

[5]Centro de Integração de Dados e Conhecimentos para a Saúde (CIDACS), Instituto Gonçalo Moniz, Fundação Oswaldo Cruz (Fiocruz - Bahia), Salvador, Brazil

[#]These authors have contributed equally to this work and share first authorship
[†]These authors have contributed equally to this work and share last authorship

*** Correspondence:**
DFDP (dariofd@gmail.com); PIPR (pablo.ramos@fiocruz.br)

Keywords: Carbapenem resistance, drug targeting, genome-scale metabolic models, *Klebsiella pneumoniae,* target selection.

1. Hole filling

Holes prediction

The PHF tool was used to search candidate genes to fill holes in the predicted pathways (Green and Karp, 2004). When the probability calculated by PHF for a given gene candidate was higher than 0.9 it was taken into account for the following checks, otherwise, it was ruled out.

Search for orthologs in *KpMGH* and function extrapolation using Autograph methodology

Orthology between the PHF suggested candidate genes and *KpMGH* genes was predicted by performing a Best Bidirectional Hit (Wolf and Koonin, 2012) using the Kbase webserver

(Arkin et al., 2018). If the functional annotation of the orthologous gene found in *KpMGH* matched the function of the reaction hole, then the function was transferred.

Evidence search databases

The candidate genes proposed by PHF were also analysed by performing BLASTp search against Ecocyc and Swiss-Prot databases to establish whether these enzymes fulfill the function proposed. In addition to taking into account parameters of identity and coverage of the alignment, it was verified that there was sufficient curated evidence to establish that the candidate fulfilled such function.

2. Refinement of incomplete metabolic pathways

Once the refining process of the holes was completed, all the incomplete metabolic pathways were checked manually (i.e, those containing reactions that could not be assigned to any of the Kp13 proteins). Removal, filling or correction of pathways was evaluated following the criteria described below.

Pathway completeness

The fact that only a few enzymes are associated with a given metabolic pathway, and therefore many gaps remain, is a clue that this metabolic pathway was incorrectly predicted. Another point to consider is whether the reactions in a predicted pathway are assigned to other metabolic pathways with greater evidence (i.e they present higher completeness or experimental evidence).

Evidence of reactions assigned to metabolic pathways variants

Variants of the same metabolic pathway often share a set of reactions (and associated genes). As an example, we can cite the variants automatically predicted by Pathway Tools for the degradation of lactose: “degradation of lactose II” and “degradation of lactose III”. The "lactose degradation II" pathway includes three reactions that carry out the process of converting lactose to D-glucopyranose. However, only one gene could be associated with one of the reactions (lacZ), leaving two holes. The “lactose degradation III” pathway presents a single reaction to which the lacZ gene could be assigned. Since lacZ is the only gene associated with the lactose II degradation pathway, it was removed.

Experimental evidence of metabolic pathways and reactions in nearby species or taxonomically isolated organisms

The lack of experimental information on the presence of a metabolic pathway in *Klebsiella pneumoniae* or related species, or the knowledge that this pathway belongs to a taxonomically distant organism gives us some evidence that this pathway should be excluded in metabolic reconstruction. This information was obtained from the Metacyc database and an exhaustive bibliographic review.

Presence of holes in the models of *Klebsiella pneumoniae* MGH 78578 and *Escherichia coli* K-12 MG1655.

The AutoGraph methodology (Derrien, André, Galibert & Hitte, 2007) was used as evidence for the prediction of missing Kp13 reactions. This methodology is based on a highly curated reference metabolic model. In this work, we use MGH 78578 and Escherichia coli K-12 MG1655 models. First, orthology relationships were sought between the genomes of the reference models and KP13. Then, gene-reaction associations between the cured networks and KP13 GEM were projected. In this way, KP13-GEM was enriched from two high-quality networks of phylogenetically close organisms.

References

Arkin, A. P., Cottingham, R. W., Henry, C. S., Harris, N. L., Stevens, R. L., Maslov, S., et al. (2018). KBase: The United States Department of Energy Systems Biology Knowledgebase. *Nat. Biotechnol.* 36, 566–569.

Green, M. L., and Karp, P. D. (2004). A Bayesian method for identifying missing enzymes in predicted metabolic pathway databases. *BMC Bioinformatics* 5, 76.

Wolf, Y. I., and Koonin, E. V. (2012). A tight link between orthologs and bidirectional best hits in bacterial and archaeal genomes. *Genome Biol. Evol.* 4, 1286–1294.
