# Supplementary material for: Pathway Driven Target Selection in Klebsiella pneumoniae: Insights Into Carbapenem Exposure
Source: Front Cell Infect Microbiol. 2022 Jan 31;12:773405. doi: 10.3389/fcimb.2022.773405 (PMC8841789; doi:10.3389/fcimb.2022.773405)
Supplement: Supplementary file 2 [file Table_1.docx]

Supplementary Information file

**Pathway-driven target selection
in carbapenem-resistant *Klebsiella pneumoniae***

**Federico Serral^1#​^, Agustin M Pardo^1#​^, Ezequiel Sosa^3^, María Mercedes Palomino^2,3^_,_ Marisa F Nicolás^4^, Adrian G Turjanski^2,3^, Pablo Ivan P Ramos^*5†^, Darío Fernández Do Porto^*1,2†^**

**^1^**Instituto de Cálculo, Facultad de Ciencias Exactas y Naturales, Universidad de Buenos Aires (UBA), Buenos Aires, Argentina

^2^Facultad de Ciencias Exactas y Naturales, Departamento de Química Biológica, Universidad de Buenos Aires, Cdad. Universitaria, Pabellón II, 4 piso, Lab QB40, C1428EGA, CABA, Buenos Aires, Argentina.

^3^Instituto de Química Biológica de la Facultad de Ciencias Exactas y Naturales (IQUIBICEN), CONICET-Universidad de Buenos Aires, Buenos Aires, Argentina.

^4^Laboratório de Bioinformática (LABINFO), Laboratório Nacional de Computação Científica (LNCC), Petrópolis, Brazil

^5^Centro de Integração de Dados e Conhecimentos para a Saúde (CIDACS), Instituto Gonçalo Moniz, Fundação Oswaldo Cruz (Fiocruz - Bahia), Salvador, Brazil

^#​^These authors have contributed equally to this work and share first authorship
^†^These authors have contributed equally to this work and share last authorship

*** Correspondence:**DFDP (dariofd@gmail.com); PIPR (pablo.ramos@fiocruz.br)

# Supplementary Table S1

**Title:** Strains and corresponding accession numbers of pathogenic *K. pneumoniae* bacteria searched for orthologs with strain Kp13 using Mauve.

| **Organism** | **Strain** | **NCBI Assembly Accession Number** |
| --- | --- | --- |
| *Klebsiella pneumoniae subsp. pneumoniae* | HS11286 | GCA_000240185.2 |
| *Klebsiella pneumoniae* | ATCC BAA-2146 | GCA_000364385.2 |
| *Klebsiella pneumoniae* | JM45 | GCA_000445405.1 |
| *Klebsiella pneumoniae subsp. pneumoniae* | PittNDM01 | GCA_000733255.1 |
| *Klebsiella pneumoniae* | PMK1 | GCA_000764615.1 |
| *Klebsiella pneumoniae subsp. pneumoniae* | KPNIH29 | GCA_000784945.1 |
| *Klebsiella pneumoniae* | XH209 | GCA_000775955.1 |
| *Klebsiella pneumoniae subsp. pneumoniae* | NTUH-K2044 | GCA_000009885.1 |
| *Klebsiella pneumoniae subsp. pneumoniae* | 1084 | GCA_000294365.1 |
| *Klebsiella pneumoniae subsp. pneumoniae* | KPNIH10 | GCA_000281435.2 |
| *Klebsiella pneumoniae subsp. pneumoniae* | KPNIH1 | GCA_000281535.2 |

| *Klebsiella pneumoniae* | 500_1420 | GCA_000406765.2 |
| --- | --- | --- |
| *Klebsiella pneumoniae* | UHKPC33 | GCA_000417085.2 |
| *Klebsiella pneumoniae* | DMC1097 | GCA_000417225.2 |
| *Klebsiella pneumoniae* | UHKPC07 | GCA_000417265.2 |
| *Klebsiella pneumoniae* | 30684/NJST258_2 | GCA_000597905.1 |
| *Klebsiella pneumoniae* | 30660/NJST258_1 | GCA_000598005.1 |
| *Klebsiella pneumoniae subsp. pneumoniae* | KPNIH24 | GCA_000714675.1 |
| *Klebsiella pneumoniae subsp. pneumoniae* | KPR0928 | GCA_000717515.1 |
| *Klebsiella pneumoniae subsp. pneumoniae* | KPNIH33 | GCA_000775375.1 |
| *Klebsiella pneumoniae subsp. pneumoniae* | KPNIH32 | GCA_000775395.1 |
| *Klebsiella pneumoniae subsp. pneumoniae* | KPNIH30 | GCA_000784985.1 |
| *Klebsiella pneumoniae* | CAV1596 | GCA_001022235.1 |
| *Klebsiella pneumoniae subsp. pneumoniae* | KPNIH27 | GCA_000695935.1 |
| *Klebsiella pneumoniae subsp. pneumoniae* | ATCC 700721; MGH  78578 | GCA_000016305.1 |
| *Klebsiella pneumoniae subsp. pneumoniae* | KPNIH31 | GCA_000785005.1 |

| *Klebsiella pneumoniae* | blaNDM-1 | GCA_000739495.1 |
| --- | --- | --- |
| *Klebsiella pneumoniae subsp. pneumoniae* | Kp13 | GCA_000512165.1 |
| *Klebsiella pneumoniae subsp. pneumoniae* | ATCC 43816 KPPR1 | GCA_000742755.1 |
| *Klebsiella pneumoniae subsp. pneumoniae* | 234-12 | GCA_000981845.1 |
| *Klebsiella pneumoniae* | Kp52.145 | GCA_000968155.1 |
| *Klebsiella pneumoniae* | CG43 | GCA_000474015.1 |
| *Klebsiella pneumoniae* | CAV1344 | GCA_001022175.1 |
| *Klebsiella pneumoniae* | CAV1392 | GCA_001022035.1 |
| *Klebsiella pneumoniae* | 32192 | GCA_000807395.3 |
| *Klebsiella pneumoniae* | HK787 | GCA_000813205.1 |
| *Klebsiella pneumoniae* | 34618 | GCA_000814305.1 |
| *Klebsiella pneumoniae subsp. pneumoniae* | 1158 | GCA_000814805.1 |
