# Supplementary material for: Pathway Driven Target Selection in Klebsiella pneumoniae: Insights Into Carbapenem Exposure
Source: Front Cell Infect Microbiol. 2022 Jan 31;12:773405. doi: 10.3389/fcimb.2022.773405 (PMC8841789; doi:10.3389/fcimb.2022.773405)
Supplement: Supplementary file 3 [file Table_2.docx]

Supplementary Information file

**Pathway-driven target selection
in carbapenem-resistant *Klebsiella pneumoniae***

**Federico Serral^1#​^, Agustin M Pardo^1#​^, Ezequiel Sosa^3^, María Mercedes Palomino^2,3^_,_ Marisa F Nicolás^4^, Adrian G Turjanski^2,3^, Pablo Ivan P Ramos^*5†^, Darío Fernández Do Porto^*1,2†^**

**^1^**Instituto de Cálculo, Facultad de Ciencias Exactas y Naturales, Universidad de Buenos Aires (UBA), Buenos Aires, Argentina

^2^Facultad de Ciencias Exactas y Naturales, Departamento de Química Biológica, Universidad de Buenos Aires, Cdad. Universitaria, Pabellón II, 4 piso, Lab QB40, C1428EGA, CABA, Buenos Aires, Argentina.

^3^Instituto de Química Biológica de la Facultad de Ciencias Exactas y Naturales (IQUIBICEN), CONICET-Universidad de Buenos Aires, Buenos Aires, Argentina.

^4^Laboratório de Bioinformática (LABINFO), Laboratório Nacional de Computação Científica (LNCC), Petrópolis, Brazil

^5^Centro de Integração de Dados e Conhecimentos para a Saúde (CIDACS), Instituto Gonçalo Moniz, Fundação Oswaldo Cruz (Fiocruz - Bahia), Salvador, Brazil

^#​^These authors have contributed equally to this work and share first authorship
^†^These authors have contributed equally to this work and share last authorship

*** Correspondence:**DFDP (dariofd@gmail.com); PIPR (pablo.ramos@fiocruz.br)

**Supplementary Table 2**

Title: Comparison of statistics on Kp13-GEM before curation, after Pathway Hole Filler improvement, and after manual annotation.

|  | Holes | Incomplete  pathways | Total  pathways |
| --- | --- | --- | --- |
| Automatic GEM | 141 | 98 | 406 |
| Pathway Hole Filler | 100 | 72 | 406 |
| Manual refinement | 92 | 68 | 386 |
